# Rotten Food for Londoners, Why?

**Published:** 1917-08-04

**Authors:** 


### ROTTEN FOOD FOR LONDONERS, WHY?

Of all the immediate consequences and influences of the war a short supply of food is obviously the one which has the most prompt and direct application to the non-combatant citizen. The threat knocks at every door, and it encloses a message charged with alarm. Diplomacy may fail and fighting plans come to naught without any acute and necessary disturbance of the calm of the household. But in the danger of famine and starvation there is a personal thrust which threatens every individual and every family. This danger has been proclaimed aloud in comfortable and prosperous England. How many of us have realised its menace? In spite of the adage it has so strange a sound that it has been widely treated as fiction. Not that there has been an entire absence of response. On the contrary, statistics show beyond doubt that a large number of citizens have voluntarily reduced their food consumption. But the general habits of the community afforded a large margin for economy, and such restrictions as have been practised have not touched hardship, much less starvation. This last word, indeed, has been generally regarded as incredible, even though it has been pronounced by highly responsible voices. The general conviction has been that here as elsewhere we shall muddle through, and that while a reduction in eating and drinking is a seemly proceeding, no real danger exists. It is a latent creed of this order which explains the absence of all substantial alarm. Yet quite certainly we are not out of the wood. And even now events which are by no means impossible may make a short food supply a situation of acute national peril. The authorities who know make the announcement. But they do not create a widely convinced audience.

There is a degree of doubt whether the authorities themselves realise the dangers from food shortage. They sound, as is their duty, the note of warning, but it is not charged with the quality of imminent danger. And certain recorded events have still further weakened its menace. Rumours of these have been spread, and we get chapter and verse in a recent report issued by the Medical Officer of Health for Stepney. The particulars are highly significant and they relate to happenings of the last few months. Fish to the tune of 100 tons purchased in Rotterdam "by a representative of the Government," allowed to remain on the quay for two or three days, and then dispatched to London without any attempt to preserve it in a proper condition; bacon, worth £50,000, utilised in the manufacture of soap, though it could have been saved had proper precautions been taken; 701 tons of potatoes found on arrival to be unfit for human consumption—such are some of the experiences which prompted Dr. Thomas to write to the Ministry of Food and to make certain suggestions. The letter provoked an official acknowledgment—but bacon is still being stored under the same old conditions and in the same old place. Still further, so far as we can learn no one has been hanged for any of the above transactions, though lamp-posts are to hand and rope may be had for the asking. When we hear that some such swift fate has overtaken the authors of these crimes we shall be ready to believe that this is indeed a beleaguered country, that the submarines threaten our national existence, and that those placed in authority over us never prophesy save in accordance with knowledge.

